# Sex-Dependent Behavioral and Biochemical Alterations in a Prenatal Oral Valproic Acid Rat Model of Autism

**DOI:** 10.1021/acschemneuro.5c00754

**Published:** 2026-03-25

**Authors:** Jaqueline Dantas Neres Martins, Lisa Maria Mendes de Almeida Souza, Leonardo Yuji Nihira Alencar, Pedro Henrique Freitas de Almeida, Caio Demetrius de Lima Meireles, Sávio Monteiro dos Santos, Kely Campos Navegantes Lima, Gabriel Mesquita da Conceição Bahia, Luana Ketlen Reis Leão da Penha, Anderson Manoel Herculano Oliveira da Silva, Karen Renata Herculano Matos Oliveira, Carlomagno Pacheco Bahia, Marta Chagas Monteiro

**Affiliations:** † Neuroscience and Cellular Biology Post Graduation Program, Institute of Biological Sciences, Federal University of Pará (UFPA), Belém 66075-110, PA, Brazil; ‡ Pharmaceutical Science Post-Graduation Program, Faculty of Pharmacy, Federal University of Pará (UFPA), Belém 66075-110, PA, Brazil; § Medical School, Medical Science Institute, Federal University of Pará (UFPA), Belém 66075-110, PA, Brazil; ∥ Laboratory of Immunology, Microbiology and In Vitro Assays (LABEIM), National Institute of Science and Technology for Pharmaceutical Innovation in the Amazon (INCT-PROBIAM), Faculty of Pharmacy, Federal University of Pará (UFPA), Belém 66075-110, PA, Brazil

**Keywords:** autism spectrum disorder, valproic acid, oxidative stress, sex differences, sociability, neurotransmission

## Abstract

Autism spectrum disorder (ASD) is a multifactorial neurodevelopmental condition characterized by impaired sociability, repetitive behaviors, and communication deficits. Animal models have been instrumental in elucidating the mechanisms underlying ASD, with prenatal exposure to valproic acid (VPA) being one of the most widely validated approaches. However, most studies rely on intraperitoneal administration, which poorly reflects human exposure. Here, we investigated the effects of oral prenatal VPA exposure in Wistar rats, focusing on behavioral outcomes, biochemical alterations, and sex-dependent differences. Pregnant females received VPA (500 mg/kg) by gavage on gestational days 11–13, and offspring were monitored from neonatal to juvenile stages. VPA-exposed pups exhibited delayed physical maturation, including postponed eye opening, tooth eruption, and locomotor development, along with reduced body weight gain. In the juvenile phase, VPA impaired sociability, reduced exploratory activity, and increased repetitive self-grooming. Importantly, behavioral effects were sex-specific: males showed more pronounced deficits in social interaction, whereas females exhibited stronger stereotyped and anxiety-like behaviors. Biochemical assays revealed elevated malondialdehyde (MDA) and nitrite levels, consistent with oxidative and nitrosative stress, especially in the hippocampus and PFC. Additionally, VPA-exposed females showed a marked reduction in hippocampal glutathione (GSH), while males exhibited increased glutamate and γ-aminobutyric acid (GABA) levels in the PFC, indicating disrupted excitatory/inhibitory balance. Collectively, our findings demonstrate that oral VPA administration induces autism-like phenotypes and region-specific neurochemical alterations in a sex-dependent manner. This study reinforces the translational validity of the oral VPA model and identifies oxidative stress and neurotransmitter imbalance as potential biomarkers for ASD pathophysiology and therapeutic intervention.

## Graphical abstract

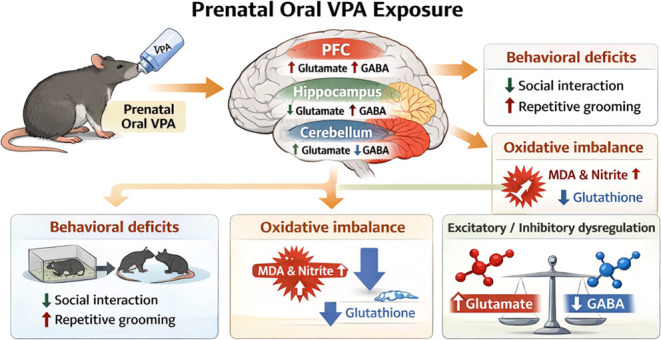

## Introduction

Autism spectrum disorder (ASD) is a multifactorial neurodevelopmental condition characterized by impairments in social communication, reduced sociability, repetitive behaviors, and cognitive alterations. Its prevalence has risen globally, affecting approximately 1 in 100–150 children, underscoring the need for translational models capable of bridging molecular mechanisms with clinical phenotypes.^1^ Among available experimental systems, prenatal exposure to valproic acid (VPA) remains one of the most robust and reproducible paradigms for modeling autism-like features in rodents.^2^

VPA is an antiepileptic and mood-stabilizing drug with nearly complete oral bioavailability.^3^ Maternal use during pregnancy is strongly associated with teratogenic and neurodevelopmental outcomes, including increased ASD risk, leading to its FDA Category D classification.^4,5^ Clinically, children exposed to VPA in utero often present cognitive impairments and higher ASD prevalence, reinforcing the translational relevance of this model.^6,7^ In rodents, prenatal oral VPA exposure induces a wide range of autism-like phenotypes—including impaired sociability, stereotyped behaviors, reduced exploratory activity, and developmental delays—providing a powerful framework for linking behavioral abnormalities with underlying neurobiological mechanisms.^2,8^

Beyond behavioral alterations, converging evidence identifies oxidative and nitrosative stress as central biochemical hallmarks of ASD. Elevated Reactive Oxygen Species (ROS), malondialdehyde (MDA), and nitric oxide (NO) are commonly reported in VPA-exposed brains, reflecting lipid peroxidation, membrane destabilization, and oxidative injury.^9,10^ Simultaneously, antioxidant defenses are compromised, with depletion of glutathione (GSH) and reduced activity of superoxide dismutase (SOD) and catalase (CAT).^11,12^ These redox alterations impair mitochondrial function, disrupt synaptic plasticity, and promote apoptotic pathways, providing a mechanistic link between oxidative imbalance and ASD-like neurological deficits.^13^ Moreover, oxidative dysregulation interacts with neurotransmitter systems: excessive glutamate levels enhance excitotoxicity, while γ-aminobutyric acid (GABA) dysfunction contributes to excitatory–inhibitory (E/I) imbalance—one of the core neurobiological mechanisms implicated in ASD.^14,15^

Recent studies also highlight sex-dependent neurobiological responses to prenatal VPA exposure. Male offspring frequently exhibit greater vulnerability to social deficits and oxidative disturbances in the prefrontal cortex (PFC), whereas females often display enhanced stereotyped behaviors and altered antioxidant capacity in the hippocampus.^16,17^ These dimorphic patterns mirror clinical observations in ASD, emphasizing the importance of evaluating biochemical and behavioral outcomes in both sexes.^18^

In this study, we selected the PFC, hippocampus, and cerebellum for detailed neurochemical and redox analyses because these regions constitute key hubs of ASD-related neuropathology. The PFC is critically involved in sociability, executive function, cognitive flexibility, and anxiety regulation, domains consistently disrupted in ASD and in VPA-exposed rodents.^19,20^ The hippocampus plays a central role in emotional regulation, learning and memory, and maintenance of glutamate/GABA homeostasis, and is highly vulnerable to VPA-induced oxidative stress, altered plasticity, and neurotransmitter imbalance.^21,22^ The cerebellum is one of the most consistently affected structures in ASD, showing Purkinje cell loss, disrupted inhibitory circuitry, and developmental dysregulation in both clinical and preclinical studies.^23,24^ Together, these regions comprise a fronto-limbic-cerebellar network mechanistically linked to ASD-like behavioral phenotypes, providing a coherent and biologically grounded framework for investigating VPA-induced neurodevelopmental alterations.

Thus, evaluating the effects of prenatal oral VPA exposure on behavior, oxidative stress, and neurotransmitter imbalance in both sexes offers an opportunity to identify mechanistic biomarkers with potential diagnostic and therapeutic relevance. By integrating behavioral phenotypes with biochemical and neurochemical end points, this study contributes to a translational understanding of ASD pathophysiology.^25^

## Experimental Section

### Animal Model and Ethical Approval

A total of 20 Wistar rats (*Rattus norvegicus*)—4 males and 16 females—were obtained from the Central Animal Facility of the Evandro Chagas Institute (IEC). All procedures were conducted in accordance with CEUA protocol No. 5503310823 and complied with the Brazilian National Council for the Control of Animal Experimentation (CONCEA) guidelines. Upon arrival, animals were acclimated for 10 days in polypropylene cages with wood-shaving bedding, maintained at 22 ± 2 °C, 55 ± 10% humidity, and maintained under controlled environmental conditions with a 12 h light/12 h dark cycle (lights on 7:00 AM–7:00 PM), with food and water provided ad libitum. Although animals were maintained under a conventional light/dark cycle due to facility constraints, all behavioral assessments were performed within a fixed time window (1:00 PM–5:00 PM), a methodological strategy shown to minimize circadian-related variability in rodent behavioral testing.^26,27^ In addition, animals were acclimated to the testing room for at least 60 min prior to each procedure to reduce stress-related behavioral artifacts. Estrous cycles were monitored daily by vaginal lavage, and females in proestrus or estrus were paired with males overnight. The presence of a vaginal plug or sperm-positive smear was designated as gestational day (GD) 0.5. Pregnant females were housed individually and weighed daily to monitor gestational progression.

### Induction of the Autism Model

Autism-like phenotypes were induced by prenatal exposure to sodium valproate (VPA). Commercial 500 mg tablets were pulverized, dissolved in distilled water (500 mg/mL), and adjusted to pH 7.4 with lactic acid. Pregnant females received oral gavage of VPA (500 mg/kg) or 0.9% saline (controls) on gestational days (GD) 11–13, a critical window corresponding to neural tube closure and early neurogenesis.^17^ After parturition, the litter was left undisturbed for 48 h. Beginning on postnatal day (PND) 3, pups were evaluated daily for neurodevelopmental milestones until PND 20. Animals were weaned at PND 21, and behavioral testing was conducted from PND 28 to 35. Brains were collected on PND 40 for biochemical and neurochemical analyses.

To ensure methodological rigor and prevent litter effects, offspring were sexed at birth and randomly selected from at least three different litters per treatment group. For the behavioral assays, 8 control males and 8 control females, and 12 VPA-exposed males and 12 VPA-exposed females were used, providing sufficient statistical power to detect sex-dependent differences. For biochemical and neurotransmitter analyses, 5 animals per sex per group (control males, control females, VPA males, VPA females) were included, totaling five biological replicates for each condition. Only one offspring per litter was used per assay, and individual selection was performed randomly. All animals were experimentally naïve at the time of testing (Figure 1).

**Figure 1:**
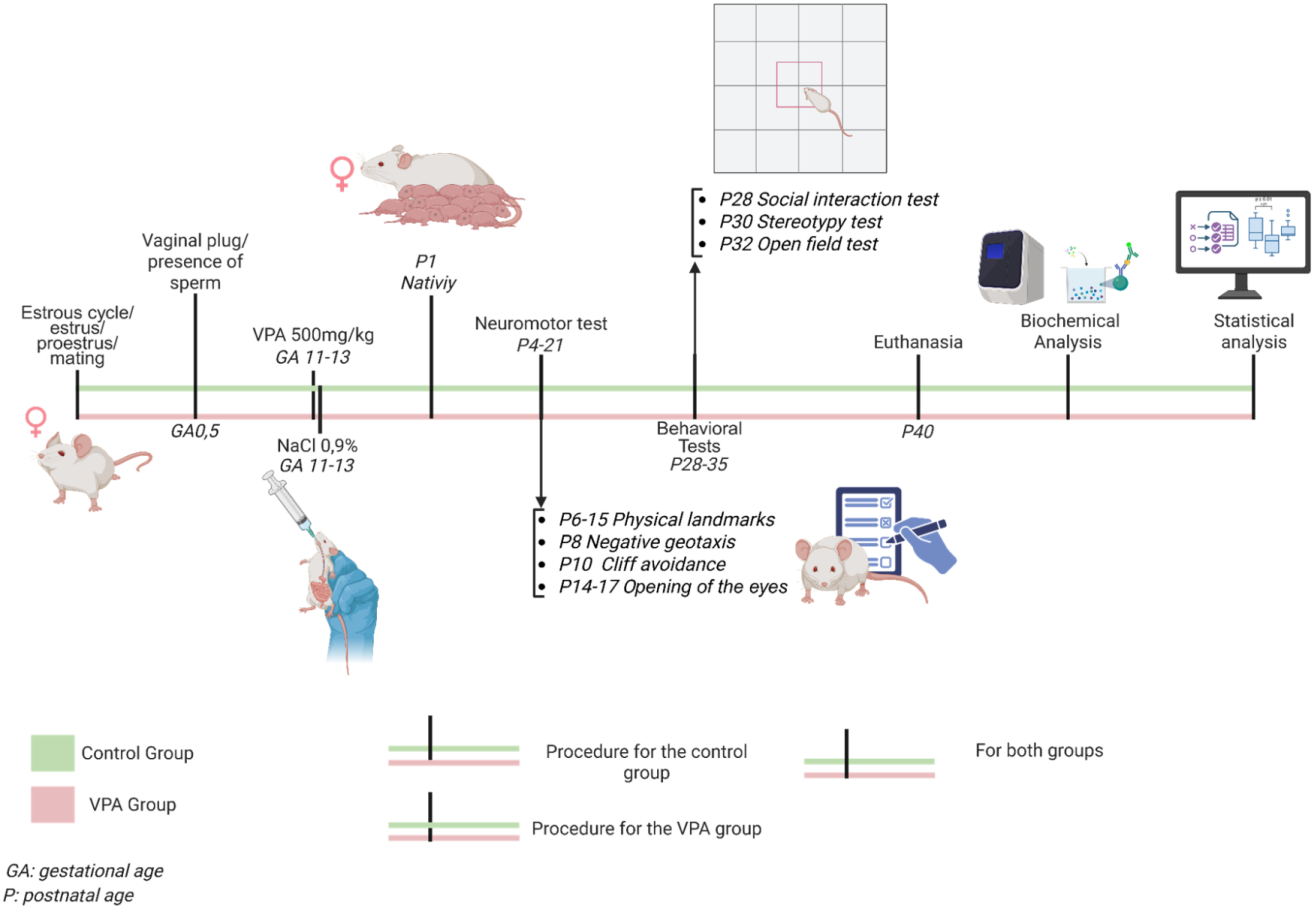
Experimental design of the VPA model of ASD. GD 0.5 was defined by the presence of spermatozoa in vaginal smears. Pregnant dams received oral gavage of VPA (500 mg/kg) or saline (CTR) on GD 11–13. The day of birth was designated as PND 1. Offspring were evaluated daily for developmental milestones from PND 3 to PND 20, followed by behavioral testing (social interaction, self-grooming, and open field) between PND 28 and 35. On PND 40, animals were euthanized for biochemical and neurochemical analyses. Behavioral assessments included CTR: 8 males, 8 females; VPA: 12 males, 12 females, while biochemical assays used *n* = 5 per sex per group.

### Neurodevelopmental Assessment

#### Physical Development

Physical maturation indices—including fur appearance, incisor eruption, gait acquisition, eye opening, and auditory canal opening—were assessed daily between PND 3 and 20. Each milestone was scored as achieved on the first day of full bilateral manifestation. Body weight was measured daily.^28^

### Sensorimotor Reflexes

- Negative Geotaxis (PND 8): Animals were placed head-down on a 45° incline, and latency to turn upward, total climb time, and number of failed attempts were recorded.^29^
- Cliff Avoidance (PND 10): Animals were placed at the edge of a 30–50 cm elevated platform. Latency to retreat was recorded as an index of sensory and motor integration maximum trial window (30 s).^26^

### Behavioral Testing

Behavioral testing was conducted under controlled conditions (22–24 °C, dim red lighting, sound isolation). Animals were acclimated to the testing room for 1 h prior to testing.^30^ All assays were video-recorded and analyzed offline by blind observers. Apparatuses were sanitized with 70% ethanol between sessions.^27^

The present study prioritized juvenile behavioral phenotyping to capture early onset neurodevelopmental and sex-dependent alterations, a developmental window that has been shown to reliably reveal ASD-like impairments in VPA models.^2,17,20^ Adult behavioral assessments (e.g., around PD60) will be incorporated in future studies to determine the developmental trajectory and persistence of these phenotypes into early adulthood.

- Social Interaction Test (PND 28): Rats were placed in an open arena (100 cm × 100 cm × 50 cm) with an unfamiliar, age- and sex-matched conspecific for 10 min. Interaction frequency, sniffing, chasing, and crossing behaviors were quantified.^24^ Videos from the Social Interaction Test were analyzed using ZebTracker software integrated with MATLAB, allowing automated extraction of social investigation time, following behavior, proximity measures, and general exploratory activity with high temporal and spatial precision. For all remaining behavioral assays, scoring was conducted manually by trained observers blinded to treatment assignment.
- Self-Grooming Test (PND 30): Rats were observed in home cages for 10 min. Frequency and duration of stereotyped grooming sequences were recorded as markers of repetitive behavior.^31^
- Open Field Test (PND 32): Rats were tested in a black Plexiglas arena (100 cm × 100 cm × 60 cm). Locomotor activity (total distance traveled), exploratory activity (entries in center vs periphery), and anxiety-like behavior were evaluated.^32^

### Biochemical Analyses

#### Tissue Collection

On PND 40, animals were euthanized under deep anesthesia, and whole brains were rapidly removed, rinsed in ice-cold PBS, and dissected into the PFC, hippocampus, and cerebellum—regions consistently implicated in ASD. Each structure was weighed, homogenized in PBS (1:20, w/v), and centrifuged at 3,000 rpm for 10 min at 4 °C. Supernatants were aliquoted and stored at −80 °C until biochemical determinations.

### Oxidative Stress Biomarkers

Oxidative stress was assessed by a comprehensive panel of biochemical markers, reflecting distinct aspects of redox imbalance and lipid/protein damage. These end points were chosen due to their translational relevance, as increased oxidative burden and reduced antioxidant defenses are consistently reported in patients with ASD.^13^

- ROS: Quantified by the H_2_DCFH-DA fluorescent assay. This probe is hydrolyzed intracellularly to DCFH, which is oxidized to the fluorescent DCF upon reaction with ROS. Elevated fluorescence indicates enhanced free radical generation, reflecting cellular redox imbalance.^33^
- GSH: Determined by Ellman’s method at 412 nm. GSH is the major intracellular antioxidant and redox buffer, and its depletion is directly linked to increased vulnerability to oxidative insults. A reduction in brain GSH is among the most consistent biochemical signatures in ASD and is considered a candidate biomarker for diagnosis and therapeutic monitoring.^34^
- Nitrite levels were determined using the Griess reaction. Excessive NO contributes to nitrosative stress, protein nitration, mitochondrial dysfunction, and excitotoxic signaling, all of which have been implicated in ASD pathogenesis.^35^
- MDA was quantified using the TBARS assay. Elevated MDA reflects peroxidative damage to polyunsaturated fatty acids in neuronal membranes, compromising membrane integrity, receptor function, and synaptic transmission.^11^

By combining these markers, the present study evaluates not only the magnitude of oxidative stress but also its biological consequences in neuronal tissues—providing a translational bridge between biochemical dysregulation and behavioral phenotypes observed in ASD.^36,37^

### Neurotransmitter Quantification (*n* = 5 per Sex per Group)

To complement oxidative stress analyses, regional tissue concentrations of the principal excitatory and inhibitory neurotransmitters, glutamate and GABA, were quantified using high-performance liquid chromatography (HPLC) with fluorescence detection following *o*-phthalaldehyde (OPA) derivatization. This approach provides high sensitivity and specificity for amino acid neurotransmitters and enables accurate quantification in discrete brain regions.^14^

### Sample Preparation

On postnatal day 40, brains were rapidly removed and dissected on ice into the PFC, hippocampus, and cerebellum. Each region was homogenized in cold phosphate-buffered saline (PBS). Homogenates were centrifuged at 12,000 × g for 10 min at 4 °C, and the resulting supernatants were deproteinized with 1% trichloroacetic acid (TCA) to precipitate proteins. After a second centrifugation step, clear supernatants were collected for derivatization and chromatographic analysis. Homoserine was used as an internal standard.

### HPLC Analysis

Samples were derivatized with OPA and *N*-acetylcysteine to generate fluorescent isoindole derivatives. The reaction mixture consisted of 10 μL of OPA and 40 μL of *N*-acetylcysteine in methanol, and a 20 μL aliquot of each derivatized sample was injected into the HPLC system.

Chromatographic separation was performed using a Shimadzu LC-20A HPLC system equipped with a fluorescence detector (FLD) and operated under LC-Solution software. Separation was achieved on a C18 reverse-phase column (250 mm × 4.6 mm, 5 μm particle size) maintained at 30 °C. A binary gradient system was employed, consisting of mobile phase A (95% sodium acetate buffer, pH 5.67, 5% methanol, and 1-propanol) and mobile phase B (70% methanol), at a flow rate of 1.0 mL/min. OPA-derivatized neurotransmitters were detected fluorometrically at an excitation wavelength of 340 nm and an emission wavelength of 455 nm.

Chromatographic peak areas were automatically integrated and normalized to the internal standard. Neurotransmitter concentrations were further normalized to total protein content determined by the Bradford method and expressed as a percentage of the sex-matched control group. This procedure quantifies total tissue neurotransmitter content.

Quantification of glutamate provided an index of excitatory neurotransmitter content, alterations of which have been associated with excitotoxic stress, mitochondrial dysfunction, disrupted synaptic plasticity, and autism-related behavioral phenotypes.^14,38^ Quantification of GABA allowed assessment of inhibitory tone; dysregulated GABA levels are strongly linked to hyperexcitability, sensory hypersensitivity, and repetitive behaviors in ASD models.^39^ The glutamate/GABA ratio was calculated as an indicator of excitatory–inhibitory (E/I) balance, a mechanistic hallmark of ASD, providing a translational bridge between oxidative stress, synaptic dysfunction, and behavioral abnormalities.^40^

### Statistical Analysis

All statistical analyses were performed using GraphPad Prism version 8.0 (GraphPad Software, San Diego, CA, USA) and Jamovi version 2.3 (The Jamovi Project, Sydney, Australia). Physical maturation milestones were compared between groups using the chi-square test, which is appropriate for categorical developmental outcomes. Behavioral and biochemical data were first assessed for normality and homogeneity of variance prior to inferential testing. For these continuous variables, a two-way analysis of variance (ANOVA) was employed, with treatment (VPA vs saline) and sex (male vs female) as independent factors. This approach allowed for the evaluation of both main effects and potential interaction effects between treatment and sex. When significant main or interaction effects were detected, Tukey’s post hoc test was applied to perform pairwise comparisons while controlling for type I error. All results are presented as mean ± standard error of the mean (SEM). Statistical significance was set at *p* < 0.05. Effect sizes (η^2^) were calculated to estimate the magnitude of treatment and sex effects where applicable, thereby providing additional information on biological relevance beyond *p*-values.

## Results

### Body Weight Development

Analysis of body weight revealed a pronounced effect of prenatal VPA exposure on somatic development. A two-way ANOVA showed a large and highly significant main effect of Treatment (*F*_1,62_ = 54.84, *p* < 0.0001, η_p_^2^ = 0.47), indicating that VPA significantly reduced offspring body weight across the postnatal period (Figure 2A). A smaller but significant main effect of Sex was also detected (*F*_1,62_ = 5.95, *p* = 0.018, η_p_^2^ = 0.09). Importantly, the Sex × Treatment interaction was not significant (*F*_1,62_ = 0.0017, *p* = 0.97, η_p_^2^ < 0.001), demonstrating that the magnitude of VPA-induced weight reduction did not differ between males and females. Effect-size estimates confirmed large reductions in both females (Cohen’s *d* = 3.46) and males (Cohen’s *d* = 1.66), indicating a substantial impact of VPA on somatic growth in both sexes. Analysis of cumulative weight gain (Figure 2B) yielded a comparable pattern. Two-way ANOVA revealed a significant main effect of Treatment (*F*_1,66_ = 38.52, *p* < 0.0001, η_p_^2^ = 0.37), confirming reduced weight gain in VPA-exposed offspring. No significant main effect of Sex was observed, and the Sex × Treatment interaction was not significant, indicating that the impairment in weight gain occurred similarly in males and females. Cohen’s d values indicated large effect sizes in both females (*d* = 1.45) and males (*d* = 1.52). Together, these findings demonstrate that prenatal oral VPA exposure produces a robust reduction in postnatal somatic growth and weight gain, independent of sex.

**Figure 2:**
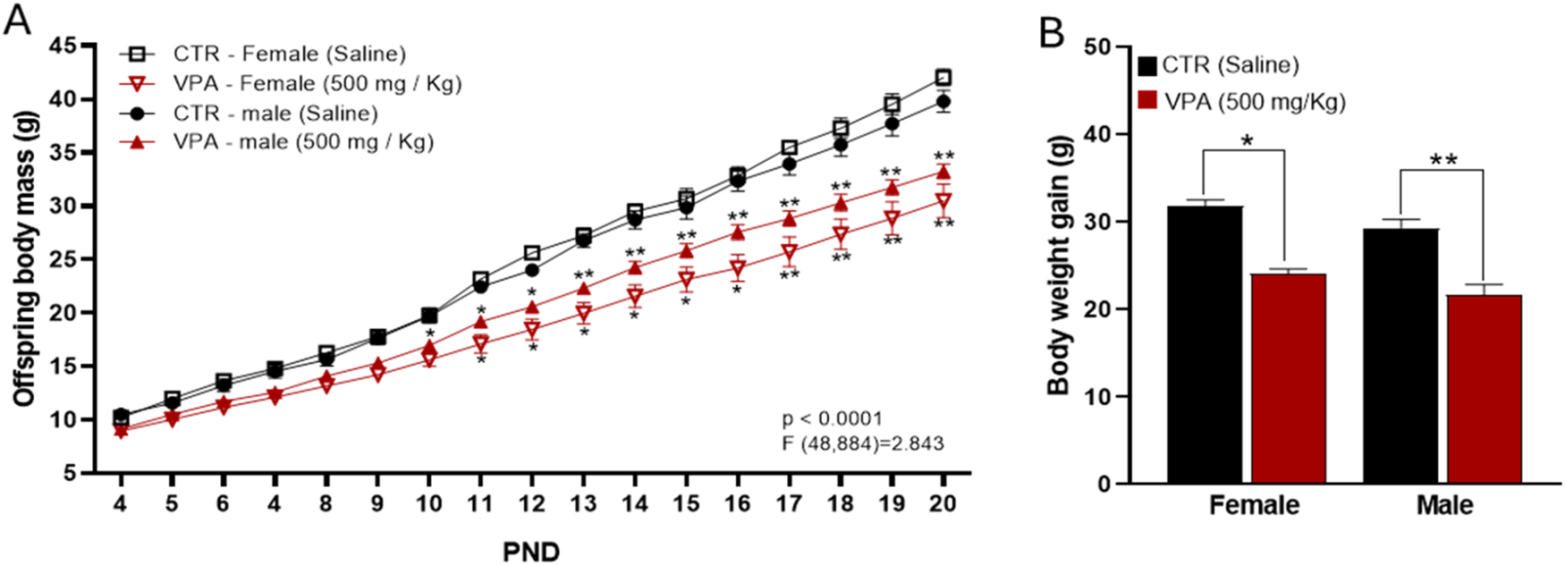
Effects of prenatal VPA (500 mg/kg) exposure on offspring body weight. (A) Longitudinal body mass measurements from PND 4 to PND 20. (B) Total body weight gain over the same period. Data are expressed as mean ± SEM. Statistical differences were evaluated against sex-matched CTR. *p < 0.05, **p < 0.001 vs CTR. Behavioral cohort sample sizes: CTR = 8 males, 8 females; VPA = 12 males, 12 females.

### Developmental Landmarks

Assessment of early physical development revealed that prenatal VPA exposure induced significant delays in several neurodevelopmental milestones in both sexes (Table 1). In control groups, fur development, upper incisor eruption, locomotor acquisition, and pinnae detachment followed expected postnatal timelines, with most pups achieving these milestones within the anticipated postnatal days (PNDs). In contrast, VPA-exposed offspring consistently exhibited delayed acquisition of these developmental landmarks. For fur development, most control females and males displayed complete coat formation by PND 6 (100% and 91.7%, respectively), whereas only 14.3% of VPA-exposed females and 5.9% of VPA-exposed males reached this milestone at the same age (χ^2^ = 32.5, *p* < 0.0001). Most VPA-exposed pups achieved fur development on PND 7, indicating a one-day delay. Upper incisor eruption was similarly delayed. All control animals exhibited eruption by PND 9, compared with only 38.1% of VPA-exposed females and 29.4% of VPA-exposed males (χ^2^ = 36.1, *p* < 0.0001), with most VPA pups reaching this milestone on PND 10. Locomotor development followed the same pattern. Whereas all control offspring displayed locomotor ability by PND 13, only 19.0% of VPA-exposed females and 17.6% of VPA-exposed males achieved this milestone at that age (χ^2^ = 34.0, *p* < 0.0001). Most VPA-exposed animals reached locomotor competence at PND 14 or later. Pinnae detachment, a sensitive marker of somatic and neural maturation, was also significantly delayed. At PND 13, all control pups had achieved this milestone, whereas only 14.3% of VPA-exposed females and 12.5% of males had done so (χ^2^ = 40.9, *p* < 0.0001). Most VPA-exposed offspring completed pinnae detachment at PNDs 15–16, corresponding to a delay of approximately two to 3 days relative to controls.^41^ Collectively, these findings demonstrate that prenatal VPA exposure produces consistent and statistically significant delays in early physical maturation across multiple developmental parameters in both sexes.

**Table 1:** Effect of Prenatal VPA (500 mg/kg) Exposure on Developmental Landmarks in Rat Pups^a^

developmental parameter	day (PND)	female control (%)	female VPA (%)	male control (%)	male VPA (%)	χ^2^
Fur development	6	100	14.3	91.7	5.9	32.5
	7	0	85.7	0	94.1	
Upper incisor eruption	9	100	38.1	100	29.4	36.1
	10	0	61.9	0	70.6	
Locomotor development	13	100	19.0	100	17.6	34.0
	14	0	76.2	0	76.5	
	15	0	4.8	0	5.9	
Pinnae detachment	13	100	14.3	100	12.5	40.9
	14	0	9.5	0	25.0	
	15–16	0	76.2	0	62.5	

a Values represent the percentage of pups achieving each developmental milestone on the indicated PND. χ^2^ values correspond to overall group comparisons between control and VPA-exposed animals for each parameter.

### Physical and Sensorimotor Development

Prenatal VPA exposure significantly affected both physical maturation and early sensorimotor reflexes in offspring (Figure 3).

**Figure 3:**
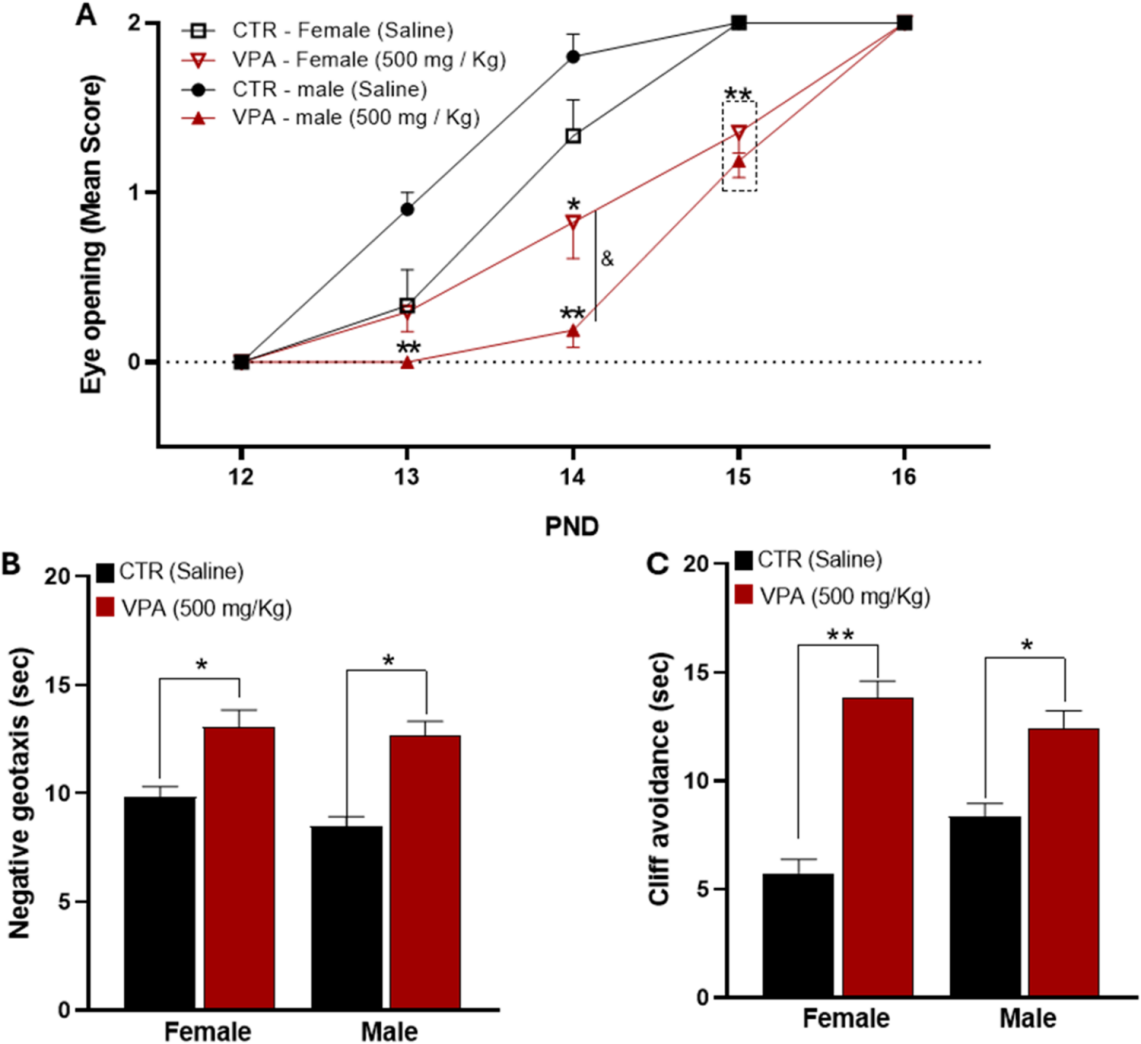
Effects of prenatal VPA (500 mg/kg) exposure on early physical and sensorimotor development in rat offspring. (A) Eye opening: percentage of pups with complete eye opening recorded from PND 12 to PND 15. (B) Negative geotaxis: latency to reorient the body on a 45° inclined surface at PND 8. (C) Cliff avoidance: latency to withdraw from the platform edge at PND 10. Data are expressed as mean ± SEM or percentage of pups achieving the developmental milestone. Statistical comparisons were made against sex-matched CTR. *p < 0.05, **p < 0.01, ***p < 0.001 vs CTR. Sample sizes for developmental assessments: CTR = 8 males, 8 females; VPA = 12 males, 12 females.

### Eye Opening

Eye opening progressed normally in control animals, with males reaching maximal scores by PND14 and females by PND15. In contrast, VPA-exposed pups exhibited a clear developmental delay, with eye-opening scores remaining significantly lower during the early and intermediate evaluation period (PND13–15). Post hoc analyses demonstrated that VPA-exposed offspring had lower mean eye-opening scores than their respective controls at PND13 and PND14, with these differences persisting into PND15. A transient sex-dependent difference was observed at PND14 within the VPA group, where males displayed lower scores than females. By PND16, all groups reached maximal values, indicating eventual convergence of maturation (Figure 3A).

### Negative Geotaxis

On PND8, control offspring rapidly reoriented when placed head-down on an inclined surface, indicating intact vestibular–motor coordination (Figure 3B). In contrast, prenatal VPA exposure significantly increased reorientation latency in both females and males. Two-way ANOVA revealed a significant main effect of Treatment (*F*_1,39_ ≈ 18.4, *p* < 0.0001, η_p_^2^ ≈ 0.32), demonstrating a robust impairment in early postural reflex performance. No significant main effect of Sex was detected (*p* ≈ 0.085), and the Sex × Treatment interaction was not significant (*p* ≈ 0.47), indicating that the magnitude of VPA-induced impairment was comparable between males and females. Post hoc comparisons confirmed that VPA-exposed females (*d* ≈ 1.31) and males (*d* ≈ 1.92) exhibited large effect sizes relative to their respective controls.

### Cliff Avoidance

On PND8, control offspring rapidly withdrew from the platform edge, reflecting intact sensorimotor integration (Figure 3C). In contrast, prenatal VPA exposure markedly increased avoidance latency in both sexes. Two-way ANOVA revealed a robust main effect of Treatment (*F*_1,30_ ≈ 94.8, *p* < 0.0001, η_p_^2^ ≈ 0.76), demonstrating a profound impairment in early defensive reflex performance. A significant main effect of Sex was also detected (*F*_1,30_ ≈ 4.52, *p* ≈ 0.041, η_p_^2^ ≈ 0.13), along with a significant Sex × Treatment interaction (*F*_1,30_ ≈ 6.17, *p* ≈ 0.018, η_p_^2^ ≈ 0.17), indicating that the magnitude of VPA-induced impairment differed between sexes. Post hoc comparisons confirmed significantly increased latencies in VPA-exposed females (*d* ≈ 3.90) and males (*d* ≈ 2.33) relative to their respective controls, with the effect being more pronounced in females. These findings indicate a strong VPA-induced disruption of early risk-avoidance behavior, with greater vulnerability in female offspring.

Taken together, these findings demonstrate that prenatal oral VPA exposure disrupts early developmental trajectories by delaying physical maturation and impairing key sensorimotor reflexes. While most effects were comparable between sexes, cliff avoidance performance revealed a sex-modulated vulnerability, with females exhibiting greater impairment. This constellation of early neurodevelopmental disturbances reinforces the translational relevance of the VPA model for investigating ASD-related developmental deficits.

### Social Behavior

Prenatal VPA exposure induced marked impairments in social interaction behaviors in both sexes (Figure 4). In control groups, males and females engaged in frequent and sustained nose-to-nose sniffing, anogenital inspection, and flank exploration, reflecting typical juvenile sociability. In contrast, VPA-exposed offspring exhibited significant reductions in both the frequency and duration of direct social investigation behaviors.

**Figure 4:**
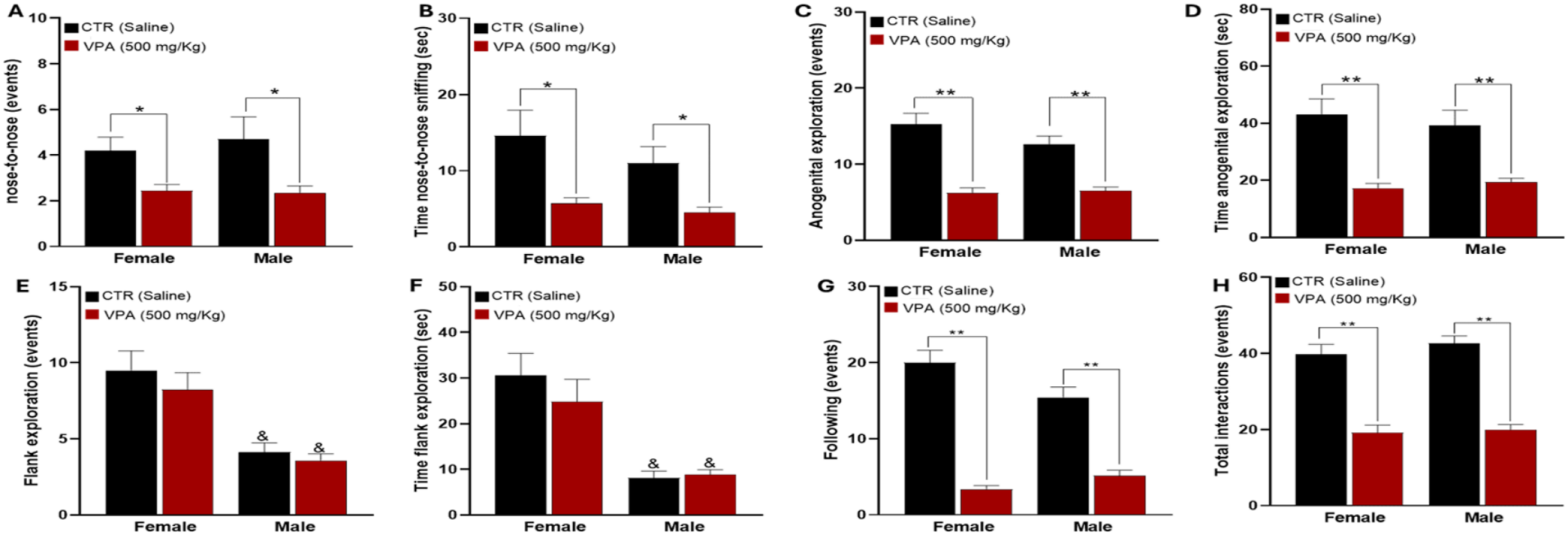
Effects of prenatal VPA (500 mg/kg) exposure on juvenile social interaction behaviors. (A, B) Frequency and duration of nose-to-nose sniffing. (C, D) Frequency and duration of anogenital inspection. (E, F) Frequency and duration of flank exploration. (G) Frequency of following behavior. (H) Total number of social interactions. Data are expressed as mean ± SEM. Statistical comparisons were made against sex-matched CTR. *p < 0.05, **p < 0.001 vs CTR. &*p* < 0.05 indicates a significant difference between VPA females and VPA males. Behavioral cohort sample sizes: CTR = 8 males, 8 females; VPA = 12 males, 12 females.

Specifically, both the number and time of nose-to-nose sniffing were significantly decreased in VPA-exposed animals, with no significant sex differences or Sex × Treatment interaction detected (Figure 4A–D), indicating comparable impairment across males and females. Similarly, the number of following behaviors—an indicator of affiliative engagement—was significantly reduced in VPA groups, resulting in an overall decrease in total social interactions (Figure 4G–H), again without evidence of sex-dependent modulation. In contrast, the behavioral parameters represented in Figure 4E,4F were not significantly affected by prenatal VPA exposure. Two-way ANOVA revealed a robust main effect of Sex in both measures, with females exhibiting higher baseline scores than males, whereas no significant main effect of Treatment or Sex × Treatment interaction was observed. These findings indicate that although certain social behaviors display clear sexual dimorphism, they are relatively resistant to VPA-induced disruption.

Collectively, these results demonstrate that prenatal oral VPA exposure selectively impairs core affiliative and direct social investigation behaviors—hallmarks of ASD-like phenotypes—while sparing other socially related domains. The pattern of reduced social exploration and affiliative engagement closely parallels clinical features of impaired sociability observed in individuals with ASD.

### Locomotor and Exploratory Behavior

The open field test performed at PND 30 revealed that prenatal VPA exposure significantly altered locomotor activity and exploration patterns in both sexes (Figure 5).

**Figure 5:**
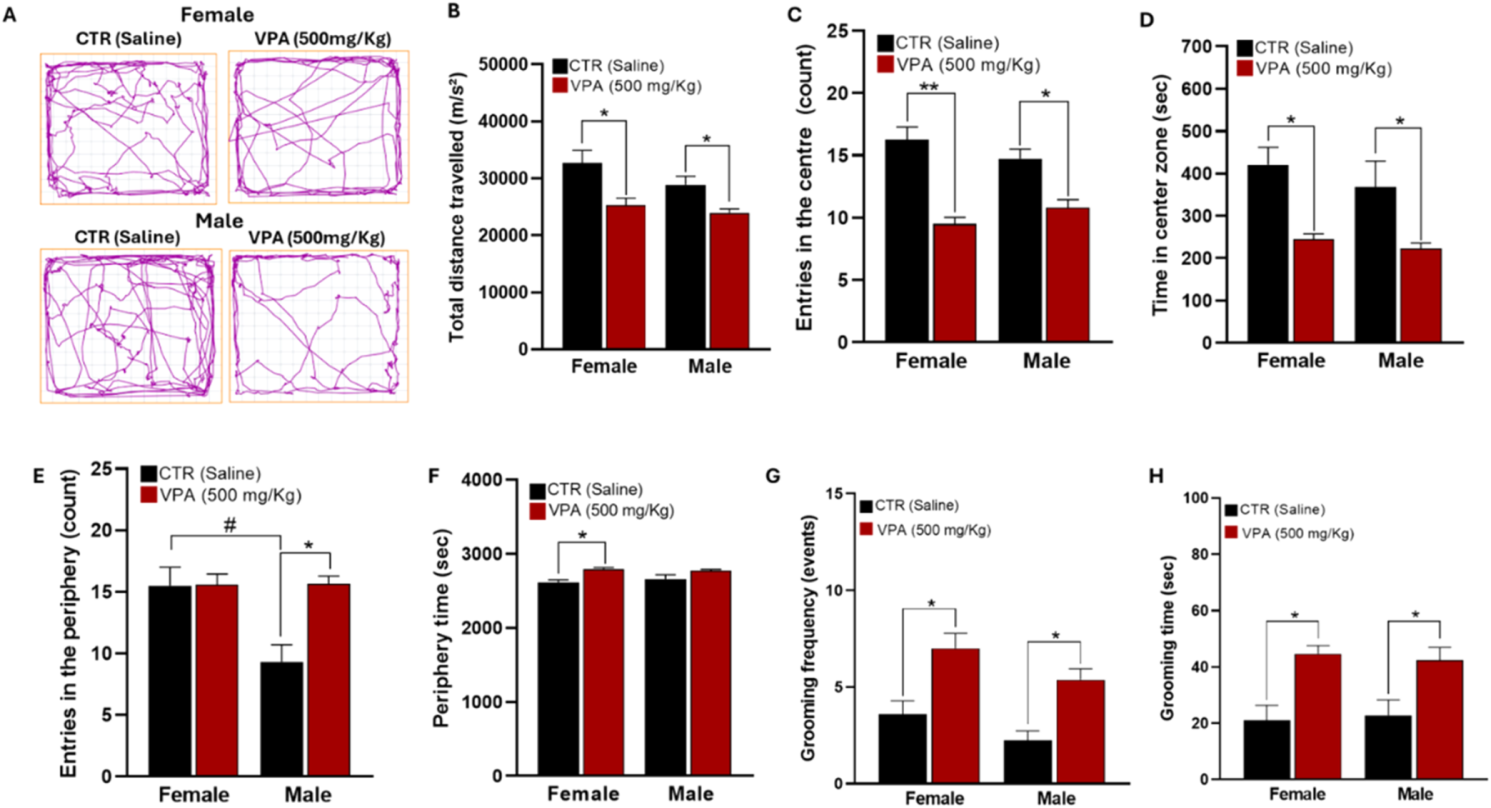
Effects of prenatal VPA (500 mg/kg) exposure on Locomotor and stereotyped behaviors at PND 30. (A) Representative locomotor traces of exploratory activity. (B) Total distance traveled. (C, D) Number of entries into, and time spent in, the center zone. (E, F) Number of entries into, and time spent in, the periphery zone. (G, H) Frequency and duration of self-grooming. Data are expressed as mean ± SEM. Statistical comparisons were made against sex-matched CTR. *p < 0.05, **p < 0.001 vs CTR. #*p* < 0.05 indicates a significant difference between CTR females and CTR males. Behavioral cohort sample sizes: CTR = 8 males, 8 females; VPA = 12 males, 12 females.

### Locomotor Activity

Representative locomotor traces demonstrated that control rats actively explored both central and peripheral areas of the arena, whereas VPA-exposed animals exhibited restricted exploration, predominantly confined to the periphery. Quantitatively, total distance traveled was markedly reduced in VPA-exposed groups compared with CTR, indicating hypoactivity and reduced exploration drive (Figure 5A–D).

### Exploratory Behavior

Control animals displayed balanced exploration of the arena, with frequent entries into and prolonged time spent in the central zone. By contrast, VPA-exposed offspring made significantly fewer entries into the center and spent less time in this zone, while exhibiting a preference for the periphery. These alterations suggest heightened anxiety-like behavior and impaired willingness to engage in open-space exploration (Figure 5E,F).

### Stereotyped Behavior

In addition to reduced locomotor activity, VPA-exposed rats exhibited increased stereotypy, as reflected by significantly elevated grooming frequency and prolonged grooming duration. Notably, these repetitive self-directed behaviors were more pronounced in females, suggesting a sex-dependent susceptibility to VPA-induced stereotypy (Figure 5G,H).

Taken together, these findings demonstrate that **prenatal oral VPA exposure induces hypoactivity, heightened anxiety-like behavior, and increased stereotyped grooming**, which collectively parallel behavioral phenotypes characteristic of ASD.

### Oxidative Stress Biomarkers

Prenatal VPA exposure induced region-specific oxidative alterations in both male and female offspring (Figure 6). Analysis of reactive oxygen species (ROS) levels revealed no significant differences between VPA-exposed and control (CTR) groups in the prefrontal cortex (PFC), hippocampus, or cerebellum (Figure 6A–C), indicating that global ROS production was not persistently elevated at the time of assessment. In contrast, lipid peroxidation was significantly increased following prenatal VPA exposure. Malondialdehyde (MDA), a stable end-product of membrane lipid oxidation, was markedly elevated in the hippocampus of VPA-exposed animals (Figure 6D–F), consistent with enhanced peroxidative damage to neuronal membranes and disruption of lipid-rich cellular structures. Nitrosative stress markers showed a partially distinct pattern. Nitrite levels were significantly elevated in the PFC of male offspring and in the hippocampus of both sexes (Figure 6G–I), suggesting increased nitric oxide production and enhanced nitrosative burden. Such alterations may contribute to mitochondrial dysfunction, synaptic instability, and excitotoxic signaling cascades. Collectively, these findings demonstrate that prenatal oral VPA exposure induces a brain region–specific oxidative imbalance characterized primarily by increased lipid peroxidation and nitrosative stress, particularly within the hippocampus. Notably, the PFC and hippocampus are critical regulators of sociability, cognition, and executive function, reinforcing the translational relevance of oxidative stress pathways as mechanistic contributors to VPA-induced ASD-like phenotypes.

**Figure 6:**
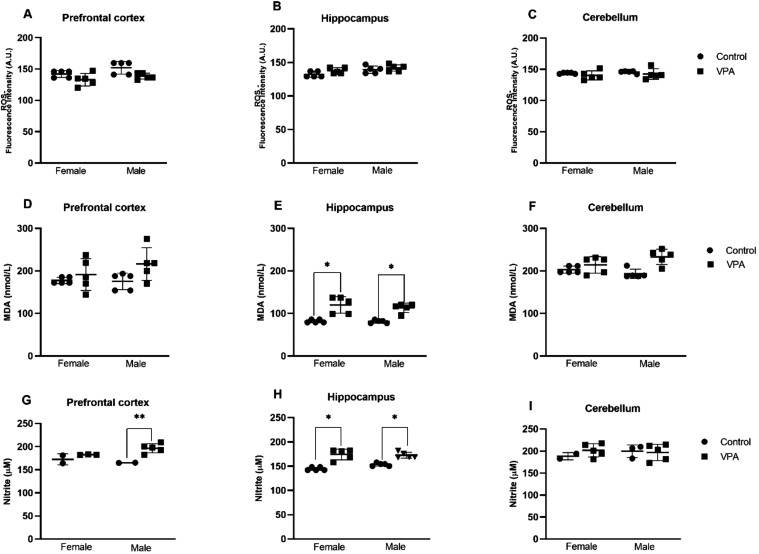
Effects of prenatal VPA (500 mg/kg) exposure on oxidative stress biomarkers in brain regions of male and female offspring. (A–C) ROS levels in the PFC, hippocampus, and cerebellum. (D–F) MDA concentrations in the same regions. (G–I) Nitrite levels in the PFC, hippocampus, and cerebellum. Data are expressed as mean ± SEM. Statistical comparisons were made against sex-matched CTR. *p < 0.05, **p < 0.001 vs CTR. Biochemical analyses were performed using *n* = 5 animals per sex per group.

### Antioxidant Defense

Analysis of reduced glutathione (GSH) levels revealed a marked region- and sex-specific pattern of susceptibility to prenatal VPA exposure (Figure 7). In the hippocampus, a highly significant Sex × Treatment interaction (*F*_1,16_ = 18.51, *p* = 0.000547, η_p_^2^ = 0.536) indicated that VPA exposure produced a pronounced reduction in GSH levels selectively in female offspring, whereas males showed no significant change relative to controls (Figure 7B). This finding demonstrates a female-specific vulnerability of hippocampal redox homeostasis. A complementary pattern was observed in the cerebellum. Two-way ANOVA revealed a significant main effect of Treatment (*F*_1,11_ = 10.99, *p* = 0.0069, η_p_^2^ = 0.50) along with a significant Sex × Treatment interaction (*F*_1,11_ = 10.44, *p* = 0.0080, η_p_^2^ = 0.49). Post hoc analyses showed that male offspring exhibited a significant decrease in cerebellar GSH levels following VPA exposure, whereas females remained unaffected (Figure 7C). This male-selective cerebellar deficit contrasts with the female-selective hippocampal reduction, highlighting a clear sex-dependent dissociation in regional redox vulnerability. In contrast, no significant effects of Sex (*F*_1,16_ = 0.020, *p* = 0.889, η_p_^2^ = 0.0013), Treatment (*F*_1,16_ = 0.930, *p* = 0.349, η_p_^2^ = 0.055), or Sex × Treatment interaction (*F*_1,16_ = 0.856, *p* = 0.369, η_p_^2^ = 0.051) were detected in the PFC (Figure 7A), indicating preserved cortical GSH levels across experimental groups.

**Figure 7:**
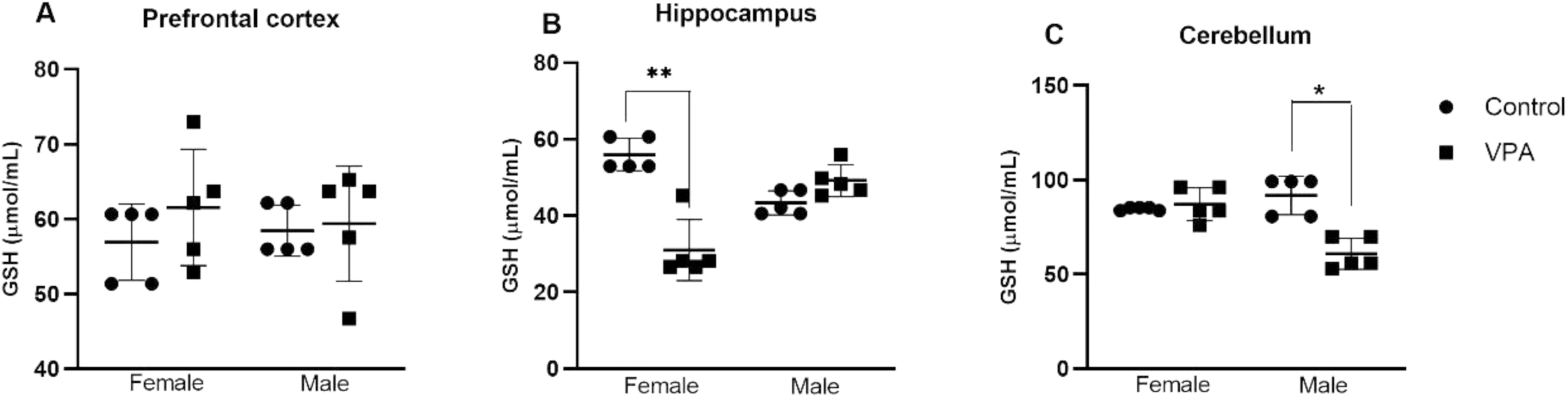
Effects of prenatal VPA (500 mg/kg) exposure on reduced glutathione (GSH) levels in offspring brain regions. (A) PFC. (B) Hippocampus. (C) Cerebellum. Data are expressed as mean ± SEM. Statistical comparisons were made against sex-matched CTR. *p < 0.05, **p < 0.001 vs CTR. Biochemical analyses were performed using *n* = 5 animals per sex per group (CTR-male, CTR-female, VPA-male, VPA-female).

Collectively, these results demonstrate that prenatal oral VPA exposure induces selective, sex-dependent reductions in GSH, characterized by hippocampal vulnerability in females and cerebellar vulnerability in males, while sparing the PFC. This region-specific pattern suggests that oxidative imbalance in the VPA model is circuit-dependent rather than global, supporting GSH depletion as a sensitive marker of targeted neurodevelopmental disruption.

### Neurotransmitter Level (Glutamate and GABA)

Prenatal VPA exposure produced marked and sexually divergent alterations in excitatory and inhibitory neurotransmission, reflected by region-specific changes in glutamate and γ-aminobutyric acid (GABA) concentrations (Figure 8). Rather than inducing a uniform directional shift, VPA generated distinct neurochemical signatures across cortical, limbic, and cerebellar circuits, resulting in a complex and sex-dependent remodeling of excitatory–inhibitory (E/I) balance.

**Figure 8:**
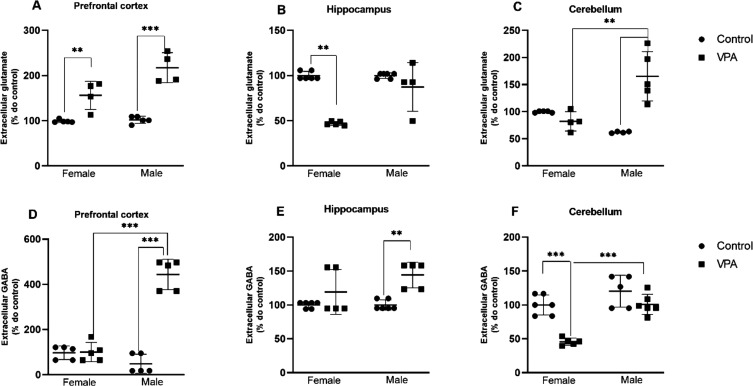
Effects of prenatal VPA (500 mg/kg) exposure on glutamate and GABA concentrations in offspring brain regions. (A–C) Glutamate levels in the PFC, hippocampus, and cerebellum. (D–F) GABA levels in the PFC, hippocampus, and cerebellum. Data are expressed as mean ± SEM. Statistical comparisons were performed against sex-matched CTR. *p < 0.05, **p < 0.001 vs CTR. Neurochemical analyses were performed using *n* = 5 animals per sex per group (CTR-male, CTR-female, VPA-male, VPA-female).

In the prefrontal cortex (PFC), VPA exposure increased glutamate levels in both sexes (Figure 8A), indicating enhanced cortical excitatory drive. However, GABAergic signaling displayed a pronounced sex-dependent divergence (Figure 8D). Female offspring showed no significant alteration in GABA levels, whereas males exhibited a dramatic and highly significant increase in GABA concentration (*t* = −11.14; *F* = 123.99; *p* = 1.4 × 10^–5^; η^2^ = 0.94; Cohen’s *d* = 7.04). Thus, in males, the PFC exhibited concomitant elevations in both glutamate and GABA levels, indicating parallel enhancement of excitatory and inhibitory signaling within cortical circuits rather than a simple excitatory shift. In the hippocampus, VPA induced a distinct pattern. Female offspring showed a significant reduction in glutamate levels (*t* = 4.88; *F* = 23.81; *p* = 0.0036; η^2^ = 0.70; Cohen’s *d* = −2.82), consistent with dampened excitatory tone (Figure 8B). In contrast, males did not exhibit a significant change in hippocampal glutamate, although greater variability was observed within the VPA group. Regarding GABA levels (Figure 8E), females showed no significant alteration, whereas males displayed a robust increase, reinforcing a hyper-GABAergic phenotype in male limbic circuitry. In the cerebellum, VPA exposure generated yet another region-specific profile. Glutamate concentrations were significantly increased in males (Figure 8C), whereas females showed no significant change. Conversely, GABA levels were significantly reduced in females (Figure 8F), consistent with impaired inhibitory regulation, while males did not exhibit significant cerebellar GABA alterations.

Collectively, these findings demonstrate that prenatal VPA exposure does not induce a generalized excitatory dominance across the brain. Instead, it produces region- and sex-dependent neurochemical reorganization characterized by concurrent elevations of glutamate and GABA in the male PFC, hippocampal glutamatergic suppression in females accompanied by male-specific inhibitory elevation, and cerebellar excitatory enhancement in males with inhibitory vulnerability in females. This circuit-specific remodeling of E/I balance aligns with contemporary mechanistic models of ASD, in which network instability arises from dysregulated coordination between excitatory and inhibitory systems rather than from a uniform neurotransmitter imbalance.

## Discussion

Prenatal oral exposure to VPA produced a constellation of neurodevelopmental alterations in rat offspring, spanning early physical development, sensorimotor and social behavior, oxidative stress, antioxidant defense, and neurotransmitter regulation, thereby providing a comprehensive model of ASD. The integration of behavioral and biochemical findings highlights not only the predictive validity of this experimental approach but also its translational relevance, since the oral route of administration better mirrors the clinical exposure pathway in pregnant women treated with valproate for epilepsy or mood disorders.^42,43^

One of the earliest manifestations of VPA exposure observed in this study was the consistent delay in physical developmental landmarks. Fur growth, incisor eruption, pinnae detachment, and eye opening were significantly postponed in VPA-exposed pups compared with CTR, and these delays were paralleled by reduced body weight gain across the first postnatal weeks. Such somatic and maturational retardation is more than a peripheral effect; rather, it reflects disruptions in neurodevelopmental trajectories, particularly in cerebellar and cortical maturation, which are strongly implicated in ASD pathophysiology. These early phenotypic markers, which are easily observable in rodent models, can be considered translational surrogates of early life developmental delays and growth deficits reported in children later diagnosed with ASD.^44,45^

The functional consequences of these maturational disturbances became evident in sensorimotor testing.^46^ VPA-exposed pups showed impaired performance in negative geotaxis and cliff avoidance tests, along with delayed eye opening, suggesting compromised vestibular, cerebellar, and cortical coordination. While negative geotaxis impairment occurred similarly in both sexes, cliff avoidance revealed a sex-modulated vulnerability, with females exhibiting greater latency increases. These deficits resonate with clinical findings of motor delay and hypotonia in children with ASD, further strengthening the translational value of these end points.^2,8^

Behavioral phenotyping in adolescence revealed marked autism-like traits. Social interaction analyses demonstrated that VPA-exposed offspring engaged significantly less in nose-to-nose sniffing, anogenital inspection, flank exploration, and following behaviors, resulting in a striking reduction in overall sociability. These impairments closely parallel the social withdrawal and reduced affiliative behavior characteristic of ASD patients. In parallel, open field testing revealed reduced locomotor activity, avoidance of the central zone, and increased stereotyped grooming, highlighting a profile of hypoactivity, heightened anxiety, and repetitive self-directed behavior. These domains correspond to the diagnostic triad of ASD: impaired sociability, increased anxiety, and stereotypy, and they replicate core deficits observed in other validated VPA models as well as in clinical populations. Of note, sex-dependent differences were again evident, with males exhibiting more profound social deficits and females showing enhanced stereotypy, echoing clinical reports of sex-dimorphic ASD phenotypes.^25,47^

Ultrasonic vocalizations (USVs), an informative measure of early communicative development in rodent ASD models, could not be assessed due to the unavailability of specialized recording equipment during the experimental period. However, because the present study focused on juvenile behavioral and neurochemical end points—developmental stages in which social interaction, exploratory dynamics, anxiety-like features, and repetitive behaviors are considered highly reliable indicators of ASD-like phenotypes—the absence of USVs does not compromise the interpretability of the behavioral findings. Future studies from our group will incorporate USV recordings to complement the developmental characterization of this model.

At the biochemical level, the convergence of oxidative stress and neurotransmitter imbalance provides mechanistic insight into the behavioral alterations observed. Although global ROS levels were not significantly altered in the analyzed brain regions, prenatal VPA exposure produced clear evidence of oxidative and nitrosative imbalance. Lipid peroxidation was significantly elevated in the hippocampus, as reflected by increased MDA levels, while nitrite concentrations were increased in the hippocampus of both sexes and in the PFC of male offspring. These alterations indicate enhanced peroxidative damage and nitric oxide–related signaling disturbances, consistent with oxidative mechanisms frequently reported in ASD.^48,49^ The hippocampus emerged as a particularly vulnerable structure, reinforcing its sensitivity to VPA-induced neurotoxicity and its critical role in learning, memory, and emotional regulation. Moreover, sex-specific patterns of redox vulnerability were observed at the antioxidant level, with hippocampal glutathione depletion occurring selectively in females and cerebellar glutathione reduction observed in males, suggesting differential regional susceptibility to oxidative insults.^50^

Neurotransmitter assays further revealed a profound disruption in excitatory–inhibitory balance. Rather than a uniform increase in glutamate accompanied by generalized GABA reduction across the same brain regions, prenatal VPA exposure produced region-specific and sexually divergent shifts in glutamatergic and GABAergic signaling. In males, the PFC showed increased glutamate accompanied by a marked elevation in GABA, consistent with a neurochemical state of concurrent excitatory upregulation and inhibitory overcompensation.

In the hippocampus, females exhibited a significant reduction in glutamate levels, whereas males displayed increased GABA concentrations without significant glutamatergic changes. In the cerebellum, VPA exposure increased glutamate levels selectively in males, while females exhibited reduced GABA levels, indicating impaired inhibitory regulation within cerebellar circuits. Together, these findings indicate circuit-dependent dysregulation of E/I tone rather than a homogeneous shift toward excitatory dominance. This imbalance is widely recognized as a mechanistic hallmark of ASD and provides a unifying link between oxidative stress, synaptic dysfunction, and behavioral deficits.^51,52^

Elevated glutamatergic signaling, particularly in cortical circuits, can contribute to excitotoxic stress and synaptic disruption and may exacerbate oxidative damage, reinforcing a vicious cycle that sustains ASD-like phenotypes in VPA-exposed animals. Notably, nitric oxide has been shown to directly modulate glutamatergic transmission and synaptic plasticity in ASD models, indicating a bidirectional crosstalk between nitrosative stress pathways and excitatory neurotransmission.^53^ Region-specific reductions in GABAergic tone, such as those detected in the cerebellum of female offspring, may impair inhibitory control and disrupt circuit homeostasis, while paradoxical hyper-GABAergic responses, as observed in the male PFC and hippocampus, may reflect maladaptive compensatory mechanisms that also destabilize network function and behavioral regulation. These findings are highly consistent with magnetic resonance spectroscopy studies in ASD patients, which reveal altered glutamate/GABA ratios in cortical and subcortical regions, providing strong translational alignment.^54,55^

Importantly, our data also converges with recent preclinical studies using the VPA model, in which combined behavioral and neurochemical assessments have been used to characterize ASD-like phenotypes. Altered expressions of glutamatergic and GABAergic genes, hippocampal glutamate dysregulation, disruption of the developmental GABA switch, and modulation of oxidative and neurotransmitter pathways have all been reported in VPA-exposed rodents and linked to sociability deficits, anxiety-like behavior, cognitive impairment, and repetitive behaviors.^56–61^ In this context, our region-level glutamate/GABA measurements, interpreted alongside oxidative stress markers and behavioral outcomes, provide a translationally relevant first-layer characterization of E/I imbalance in the oral VPA model. Future studies will build upon these findings by incorporating synapse-resolved and cell-type-specific approaches to refine the circuit-level understanding of these neurochemical alterations.

The neurochemical profile revealed in this study aligns closely with evidence that prenatal VPA exposure disrupts the maturation of excitatory and inhibitory circuits through mechanisms that are both regionally selective and sexually divergent. Consistent with our findings of hippocampal glutamatergic suppression in females and cerebellar inhibitory vulnerability, previous studies have shown that VPA impairs excitatory synaptogenesis and reduces interneuron density in hippocampal and cerebellar circuits, particularly affecting Purkinje cells and inhibitory microcircuits central to motor and socio-affective processing.^62,63^ Conversely, the cortical hyperglutamatergia and marked GABAergic overcompensation observed in males parallel reports of altered cortical maturation in VPA-based models, including increased glutamate levels, altered GAD67 expression, and dysregulated vesicular GABA/glutamate transport.^21,64^ Such sex-specific neurochemical signatures are consistent with clinical observations that autism exhibits a male-biased E/I imbalance, potentially arising from differential sensitivity to early life insults that affect interneuron migration, synaptic pruning, mitochondrial metabolism, and neuroinflammatory cascades.^65,66^ Taken together, these data reinforce the concept that VPA does not shift excitation or inhibition uniformly, but instead generates divergent, circuit-dependent trajectories—ranging from excitatory dampening to paradoxical inhibitory overdrive—that recapitulate core mechanistic hallmarks of ASD.^66–69^

The integration of behavioral, biochemical, and neurochemical findings presented here underscores the utility of the oral VPA model as a translational platform for ASD research. Unlike intraperitoneal administration, which produces robust but often exaggerated systemic toxicity, the oral route more closely mimics clinical exposure while still reproducing the full spectrum of ASD-like phenotypes. This enhances its translational relevance, particularly for evaluating therapeutic interventions aimed at modulating oxidative stress or restoring excitatory–inhibitory balance. The identification of convergent biomarkers—including delayed developmental milestones, reduced growth, impaired sociability, increased stereotypy, elevated MDA and nitrite levels, glutathione depletion, and sex-dependent alterations in glutamate/GABA signaling—provides a multidimensional framework for preclinical screening of pharmacological and nutraceutical candidates. Importantly, the demonstration of pronounced sex differences across behavioral, redox, and neurotransmitter domains further emphasizes the necessity of incorporating sex as a biological variable, in line with current NIH guidelines and clinical observations of differential ASD presentation between males and females.

In conclusion, prenatal oral VPA exposure recapitulates the defining features of ASD at multiple biological levels—from early developmental delays and altered socio-exploratory behavior to oxidative imbalance and neurotransmitter dysregulation. The convergence of these findings establishes a strong mechanistic bridge between teratogen-induced neurotoxicity and long-standing neurodevelopmental dysfunction, reinforcing both the constructive and predictive validity of the oral VPA model. By linking behavioral phenotypes to quantifiable biochemical and neurochemical alterations, this study identifies oxidative stress pathways and excitatory–inhibitory imbalance as critical translational end points and promising therapeutic targets, strengthening the case for the oral VPA model as a powerful platform for advancing preclinical ASD research.

## Conclusion

Our findings demonstrate that prenatal oral exposure to VPA is sufficient to disrupt neurodevelopment across multiple domains, producing a constellation of alterations that mirror the multifactorial nature of ASD. The model consistently induced delays in physical and neurological maturation, followed in the juvenile period by reduced sociability, increased repetitive grooming, and altered exploratory behavior, several of which displayed sex-dependent trajectories.

Beyond behavioral phenotypes, VPA exposure produced region-specific disturbances in redox homeostasis. Although global ROS levels were not significantly altered, VPA increased lipid peroxidation in the hippocampus and elevated nitrite levels in the hippocampus and male prefrontal cortex, indicating enhanced oxidative and nitrosative stress signaling. In parallel, antioxidant capacity exhibited sexually divergent vulnerability, with hippocampal glutathione depletion occurring selectively in females and cerebellar glutathione reduction detected in males. Together, these findings highlight oxidative imbalance as an important contributor to neurodevelopmental vulnerability in this model.

Crucially, neurochemical analyses revealed marked disturbances in excitatory–inhibitory balance across brain regions. Females exhibited significant suppression of hippocampal glutamatergic signaling together with reduced cerebellar GABA levels, whereas males showed prefrontal hyperglutamatergia accompanied by pronounced increases in GABA in both the prefrontal cortex and hippocampus, as well as elevated cerebellar glutamate levels. These sexually divergent neurochemical patterns suggest circuit-specific dysregulation of excitatory and inhibitory signaling and provide mechanistic insight into the biological heterogeneity observed in ASD.

Taken together, these results position the oral VPA model as a robust and translationally relevant paradigm capable of integrating developmental, behavioral, oxidative, and neurochemical outcomes into a unified mechanistic framework. Unlike intraperitoneal protocols, which can exaggerate systemic toxicity, the oral route more closely reflects clinical exposure conditions, thereby strengthening the construct and predictive validity of the model. The convergence of ASD-like behavioral phenotypes with quantifiable biochemical and neurotransmitter alterations highlights the potential of this approach for identifying region- and sex-specific biomarkers and for evaluating therapeutic strategies aimed at restoring redox balance and normalizing excitatory–inhibitory signaling. In summary, prenatal oral VPA exposure provides a powerful and clinically relevant platform for advancing ASD research. By bridging mechanistic neurobiology with therapeutic discovery, this model offers important opportunities to refine translational biomarkers and to guide the development of interventions targeting circuit-specific and sex-dependent vulnerabilities in autism spectrum disorder.

## Abbreviations

ASD: autism spectrum disorder
VPA: valproic acid
PFC: prefrontal cortex
GSH: reduced glutathione
ROS: reactive oxygen species
MDA: malondialdehyde
NO: nitric oxide
GABA: γ-aminobutyric acid
E/I: excitatory–inhibitory
PND: postnatal day
GD: gestational day
HPLC: high-performance liquid chromatography
OPA: *o*-phthalaldehyde
TCA: trichloroacetic acid
PBS: phosphate-buffered saline
ANOVA: analysis of variance
SEM: standard error of the mean
TBARS: thiobarbituric acid reactive substances
USVs: ultrasonic vocalizations
